# Evaluation of Arbuscular Mycorrhizal Fungi Capacity to Alleviate Abiotic Stress of Olive (*Olea europaea* L.) Plants at Different Transplant Conditions

**DOI:** 10.1155/2014/378950

**Published:** 2014-02-12

**Authors:** María Josefina Bompadre, Mariana Pérgola, Laura Fernández Bidondo, Roxana Paula Colombo, Vanesa Analía Silvani, Alejandro Guillermo Pardo, Juan Antonio Ocampo, Alicia Margarita Godeas

**Affiliations:** ^1^Departamento de Biodiversidad y Biología Experimental, Facultad de Ciencias Exactas y Naturales, Universidad de Buenos Aires, Intendente Güiraldes 2160, Ciudad Universitaria, 4^to^ Piso, Pabellón 2, C1428EGA Buenos Aires, Argentina; ^2^Laboratorio de Micología Molecular, Departamento de Ciencia y Tecnología, Universidad Nacional de Quilmes, Roque Sáenz Peña 352 Bernal, B1876BXD Buenos Aires, Argentina; ^3^Departamento de Microbiología del Suelo y Sistemas Simbióticos, Estación Experimental del Zaidín, CSIC, Profesor Albareda 1, 18008 Granada, Spain

## Abstract

The capacity of roots to sense soil physicochemical parameters plays an essential role in maintaining plant nutritional and developmental functions under abiotic stress. These conditions generate reactive oxygen species (ROS) in plant tissues causing oxidation of proteins and lipids among others. Some plants have developed adaptive mechanisms to counteract such adverse conditions such as symbiotic association with arbuscular mycorrhizal fungi (AMF). AMF enhance plant growth and improve transplant survival by protecting host plants against environmental stresses. The aim of this study was to evaluate the alleviation of transplanting stress by two strains of *Rhizophagus irregularis* (GC2 and GA5) in olive. Our results show that olive plants have an additional energetic expense in growth due to an adaptative response to the growing stage and to the mycorrhizal colonization at the first transplant. However, at the second transplant the coinoculation improves olive plant growth and protects against oxidative stress followed by the GA5-inoculation. In conclusion, a combination of two AMF strains at the beginning of olive propagation produces vigorous plants successfully protected in field cultivation even with an additional cost at the beginning of growth.

## 1. Introduction

Roots are highly sensitive to soil physicochemical parameters [1, 2]. Under abiotic stress they can generate ROS such as singlet oxygen, superoxide, hydrogen peroxide, and hydroxyl radicals [3, 4]. When accumulation of ROS exceeds the removing capacity of the antioxidant system they cause important oxidative damage to proteins, lipids, and photosynthetic pigments as well as inactivation of photosynthetic enzymes [5]. Plants can detoxify these oxidative molecules through ROS-scavenging enzymes such as catalase (CAT), superoxide dismutase (SOD), and ascorbate peroxidase (APX) [6].

Some plants have developed mechanisms to mitigate abiotic stress such as increasing their root system or associating symbiotically with AMF for a better exploration of soil and improvement of nutritional status [7]. Over 90% of plant species are associated with AMF including forest trees, wild grasses, and many crops [8]. Previous studies have demonstrated that plant inoculation with AMF improves establishment and increases biomass and survival rate under different environmental stresses [8–10]. Besides, AMF can greatly contribute to crop productivity and environmental sustainability [11].

Olive plants are propagated by semiligneous cuttings of mother plants under active vegetative growth. To prevent cutting desiccation they need a moist and cool environment. In order to achieve good rooting, temperatures between 20 and 25°C in the base of the cutting and a wet environment at the aerial part are necessary. Nursery cultivation is the most convenient way to improve the success of transplantation at the crop area [12]. Olive plants suffer at least two transplant moments in nursery conditions prior to outside cultivation. AMF can reduce transplant stress by changing the morphology of the root system favouring the establishment of plants [13, 14].

Artificial inoculation of olive plant cuttings with AMF has been adopted by an increasing number of nursery managers as a method for promoting growth, production, and precocity [15]. Moreover, it is an essential component for most plants and it can be used as a biofertilizer resource [11]. In general, abiotic stress causes extensive losses to agricultural productivity and thus the aim of this study was to evaluate the alleviation of transplanting stress in olive plants by two strains of the AM fungal species *Rhizophagus irregularis*.

## 2. Materials and Methods

### 2.1. Plants and Inocula

A vigorous and youthful mother plant of *Olea europaea *L. cv. Manzanillo 4 m in height was chosen from the Departamento de Producción Vegetal (Facultad de Agronomía, FAUBA, Argentina). Young branches (a total of 275 cuttings) of 7 cm length and 2 shoots from this only olive plant were made in order to eliminate genetic variability and to obtain healthy and vigorous cuttings. The cut base of the cutting was dipped into a hormone rooting powder made up of 2,500 ppm of indole butyric acid (IBA) dissolved in ethanol and adsorbed onto talcum powder [16].

The cuttings were rooted in perlite (1 m wide by 10 m long and 10 cm deep) on a raised table to 1 m at a 1,000 cuttings per m^2^ density in order to maintain a constant humidity, reduce temperature, and create appropriate microclimate conditions for olive rooting. Cuttings were under intermittent irrigation over a period of 60 days in nursery conditions. A 70% of rooting was obtained and provided sufficient plants for the study.

The raised rooting table was divided into 4 separated parts with plastic panels to prevent mycorrhizal and cuttings roots advance. After 30 days of rooting period, they were inoculated with two strains of *R. irregularis* (formerly *Glomus intraradices*), GC2 and GA5, which have different strategies of colonization *in vitro* and in soil conditions. These strains were provided by the Banco de Glomeromycota *In Vitro* (BGIV) (http://www.bgiv.com.ar/strains/Rhizophagus-intraradices/gc2; http://www.bgiv.com.ar/strains/Rhizophagus-intraradices/ga5). The GC2 strain has a high density of external mycelium, slow growing at the beginning of *in vitro* culture which increases with the proportion of mycelium ramification, and few number of big spores (160.52 ± 19.8 cm^2^; 87.4 ± 0.4 µm) [17]. Its spores and mycelium are limited to the vicinity of the roots where the colonization takes place. In contrast, GA5 presents little external mycelium at the beginning of culture but then increases its density forming a mycelium little branched, has a higher growth rate, and its spores are smaller and more abundant than GC2 (293.4 ± 81.8 cm^2^; 70.8 ± 0.5 µm) [17].

The two strains used were propagated in *Trifolium repens* as host in 1.5 L pots with a mixture of perlite : soil (3 : 1) sterilized by tyndallization (100°C for 1 h, three consecutive days). The soil characteristics were pH 7.1; total C 12.08 and N 1.1 g kg^−1^; P 34.2 mg kg^−1^; K 0.9, Ca 7.5, Mg 1.7, and Na 0.2 cmol kg^−1^. They were kept during four months under greenhouse conditions (450 µE·m^−2^ s^−2^, 400–700 nm; 16/8 light-darkness; 25/18°C day/night; 60–70% relative humidity). All plants were watered with Hewitt [18] solution without phosphorous addition every 15 days and thereafter they were unwatered to dryness to obtain dry general mycorrhizal inoculum.

Cuttings were inoculated as follows: control without AMF (C); inoculation with *R. irregularis *strain GC2 (GC2); inoculation with *R. irregularis *strain GA5 (GA5); and inoculation of a 1 : 1 mixture of GA5 and GC2 strains (GA5 + GC2). For inoculation, furrows were made between groups of cuttings that were 3 cm deep. A total of 10 g of dry inoculum of the appropriate strain was then added to the furrow for each treatment. It was estimated that there were for GA5: 1,161 ± 13 spores/100 g dry soil and for GC2: 851 ± 5 spores/100 g dry soil. The control treatment received 10 g of autoclaved mixture inoculum supplemented with a filtrate (<20 µm) of mycorrhizal inoculum to provide similar microbial population.

### 2.2. Experimental Design

#### 2.2.1. First Experiment

After 30 days of cuttings inoculation on the raised rooting table under nursery conditions, half of the first experiment (48 cuttings) was transplanted to 0.5 L pots with tyndallized perlite : vermiculite : soil (2 : 1 : 1) (see above for soil characteristics and tyndallization) and the other half (48 cuttings) was kept on the raised rooting table. The following samplings were made: T0: cuttings rooted in raised table and T1: 3 d after transplant in 0.5 L pots with tyndallized substrate perlite : vermiculite : soil (2 : 1 : 1). Initially pots were set to field capacity (70 ± 2%); after 3 d, pots were found at 57 ± 1.1% of field capacity. Plants were not fertilized.

#### 2.2.2. Second Experiment

For the second experiment, a total of 96 cuttings were transplanted in 1.5 L pots with a mixture of tyndallized perlite : vermiculite : soil (2 : 1 : 1). They were maintained at 70% of field capacity and were fertilized without phosphorous addition [18] every month for a period of 12 months under nursery conditions. After that half of the experiment (48 plants) was transplanted to 4 L pots with nonsterile soil (see above for soil characteristics) and irrigated with water only at the beginning of transplant. They were maintained for 7 days under nursery conditions. The other half (48 plants) was kept in 1.5 L pots in sterile soil. Two samples were taken, T2: plants in 1.5 L pots in sterile soil, and T3: elapsed 7 days after 4 L pots transplant to nonsterile soil. At the beginning of transplant, soil field capacity was 60.2 ± 1.6%, and at T3 the soil field capacity was found at 21.6 ± 4.2%. Plants were not fertilized.

### 2.3. Growing and Biochemical Parameters

Mycorrhization was tested 30 days after inoculation (T0) and before the second transplant experiment (T2). To this end a representative portion of root was stained according to [19], and quantification was made according to [20]. Measurements were discriminated as mycorrhizal percentage (MI%), arbuscules percentage (A%), and vesicles percentage (V%) [21]. From each sampling survival percentage, fresh and dry weights from shoots and roots were recorded (70°C to constant weight). Water content was calculated as the difference between fresh and dry weights. Also shoot-to-root ratio plant biomass was evaluated. Mycorrhizal dependency (MD%) was calculated as the (mycorrhizal plant biomass/nonmycorrhizal plant biomass average) ∗ 100 [22].

For the biochemical parameters measurements fresh plant material (shoots and roots) was weighed and 1 g was immediately immersed in liquid nitrogen to maintain the integrity of the tissue until use, and each sample was pulverized in a mortar with liquid nitrogen. Six mL of extraction buffer (KH_2_PO_4_-K_2_HPO_4_ 50 mM pH 7.8 plus 0.1 mM EDTA) and polyvinylpolypyrrolidone (PVPP, 0.06 g/6 mL extraction buffer) were added. The resulting mixture was filtered through a nylon membrane to remove cell debris and centrifuged at 13,000 rpm during 20 minutes. Supernatants were aliquoted and maintained at −70°C until use [23].

The following intracellular enzyme activities associated with oxidative stress were measured. Catalase (CAT) (EC 1.16.1.6): following a method based in absorbance diminishing measure at 240 nm occasioned by H_2_O_2_ disappearance [24]. Ascorbate peroxidase (APX) (EC 1.11.1.11): method based on 290 nm measure of ascorbic acid oxidation [25]; a solution of ascorbic acid (4 mM) was added to aliquots in order to preserve this enzyme activity [26]. Superoxide dismutase (SOD) (EC 1.15.1.1): determination based on superoxide dismutase capacity to inhibit nitroblue tetrazolium (NBT) reduction to superoxide radicals generated photochemically [27]. Nonenzymatic activity measurements were also quantified. Total protein content (PROT): according to [28]. Malondialdehyde content (MDA): measured by the reaction to thiobarbituric acid (TBA) according to [29]. All enzyme activities and MDA content were standardized by protein content.

### 2.4. Statistical Analysis

All data were subjected to analysis of variance (factorial ANOVA). Homogeneity of variance and normal distribution were checked. Comparisons among mean values in each treatment were made using the Tukey test (honest significant difference HSD) (*P* < 0.05) [30]. Statistical procedures were carried out with the software package STATISTICA 6.0 for Windows XP.

## 3. Results

### 3.1. First Experiment

Noninoculated plants had the highest survival percentages followed by GA5 and coinoculated ones. No differences were observed in mycorrhiza, arbuscules, and vesicles percentages in both strains single and coinoculated (Table 1).

Fresh, dry, and water contents of olive shoots were negatively affected in all treatments at the first transplant condition, and no differences were observed in roots. Shoot-to-root ratio was variable but no differences were observed. MD was affected neither by transplant nor by treatments (Table 2).

**Figure 1:**
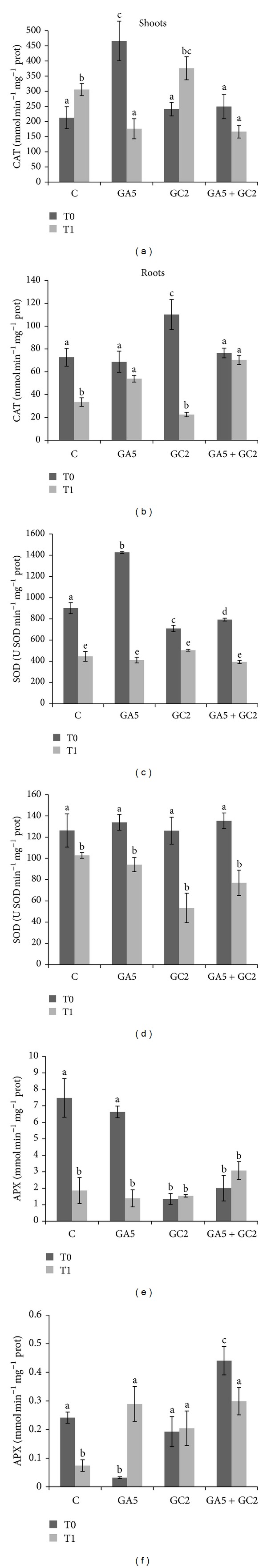
Enzyme activities in olive shoots (a, c, and e) and roots (b, d, and f). Catalase (CAT), superoxide dismutase (SOD), and ascorbate peroxidase (APX). Treatments: C (control without inoculation); GA5 (*Rhizophagus irregularis *GA5 strain inoculation); GC2 (*Rhizophagus irregularis *GC2 strain inoculation); GA5 + GC2 (mixture 1 : 1 of GA5 and GC2 strains). T0 (on raised table); T1 (after 3 d transplanted on sterile soil). Different letters indicate significant differences at *P* < 0.05. Data were analyzed with factorial ANOVA. Data represent mean of 6 replicates ± standard error.

**Figure 2:**
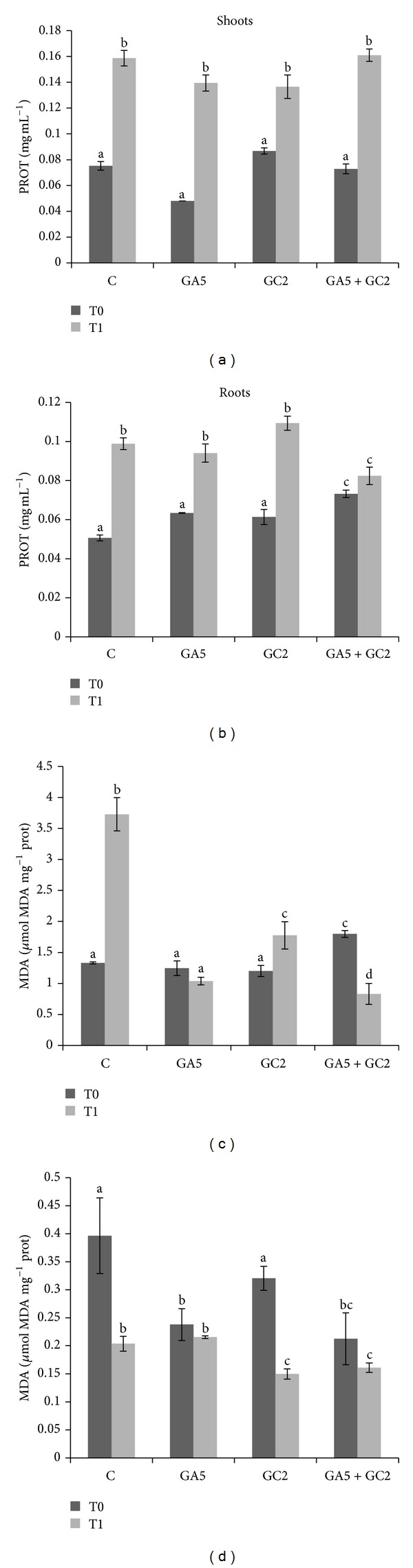
Total protein (PROT) and malondialdehyde (MDA) contents in olive shoots (a, c) and roots (b, d). Treatments: C (control without inoculation); GA5 (*Rhizophagus irregularis *GA5 strain inoculation); GC2 (*Rhizophagus irregularis *GC2 strain inoculation); GA5 + GC2 (mixture 1 : 1 of GA5 and GC2 strains). T0 (on raised table); T1 (after 3 d transplanted on sterile soil). Different letters indicate significant differences at *P* < 0.05. Data were analyzed with factorial ANOVA. Data represent mean of 6 replicates ± standard error.

A significant interaction was observed for all enzyme activities in olive shoots. Control plants and GC2-inoculated plants increased CAT activity in T1, contrary to GA5-inoculation observations. At T0 condition, GA5-inoculated plants showed higher CAT activity than the rest of the treatments, and at T1 they decreased more than control plants. Moreover, there were no changes observed in CAT enzyme activity in coinoculated olive shoots (Figure 1(a)). At T0 SOD enzyme activity was significantly higher in GA5-inoculated plants followed by control > coinoculation > GC2-inoculated plants. However, at T1 condition SOD activity decreased for all treatments (Figure 1(c)). At T0 condition control plants and GA5-inoculated plants had higher APX enzyme activity than GC2 and coinoculated plants. However, at T1 condition all APX activities were significantly lower but similar for all treatments (Figure 1(e)). PROT increased for all treatments at T1 (Figure 2(a)). The MDA content greatly increased in control plants at T1 but in GA5-inoculated plants MDA was not affected. The GC2-inoculated plants increased MDA at T1, but these contents were significantly lower than control plants. In coinoculated plants at T0 MDA content was significantly higher than control plants. However, at T1 condition these contents were significantly lower than control plants at both conditions (Figure 2(c)).

In roots, CAT enzyme activity significantly decreased in control and GC2-inoculated plants. Moreover, at T0 the GC2-inoculated plants had higher CAT activity than control plants at the same condition (Figure 1(b)). On the other hand, SOD enzyme activity decreased at T1 for all treatments (Figure 1(d)). Control plants and GA5-inoculated plants had contrary responses to APX enzyme activity; at T1 GA5-inoculated plants increased APX activity whereas control plants decrease it. The GC2-inoculated plants had similar APX enzyme activity to control plants in T0 condition. Moreover, coinoculated plants had higher APX in comparison to control plants, but at T1 condition these enzyme activities decreased at similar values to control plants at T0 condition (Figure 1(f)). PROT increased at T1 in single inoculation and control plants. On the other hand, there were no differences observed in coinoculated plants but these values were significantly higher than control plants at T0 condition (Figure 2(b)). MDA content decreased in T1 for all treatments; nevertheless, only control plants and GC2-inoculated plants had significant differences. However, at T0 condition GA5-inoculated plants and coinoculation had less MDA content than control plants (Figure 2(d)).

### 3.2. Second Experiment

One year after growing under nursery conditions olive plants had similar mycorrhizal percentages. Arbuscules and vesicles percentages were similar in GA5 and coinoculated plants. The GC2-inoculated plants had high arbuscules percentages and low vesicles percentages. Coinoculated plants survived 100%, followed by control plants (Table 3).

There was a significant improvement in shoot and root fresh weight, biomass, and water content of GA5-inoculated plants at T2 condition in comparison to control plants. Nevertheless, at T3 they decreased but these values were higher than control plants (Table 4). Coinoculated plants improved shoot and root fresh weight and biomass at T3 condition. The GC2 and coinoculated plants had less shoot and root water content at T2 condition than control plants. Under T3 condition they increased at similar values compared to GA5-inoculated plants. Moreover, these values were higher than control plants. No differences were observed in shoot-to-root ratio for all treatments and transplant conditions tested. The MD increased in shoots and roots at T3 in GC2 and coinoculated plants, contrary to GA5-inoculated plants (Table 4).

**Figure 3:**
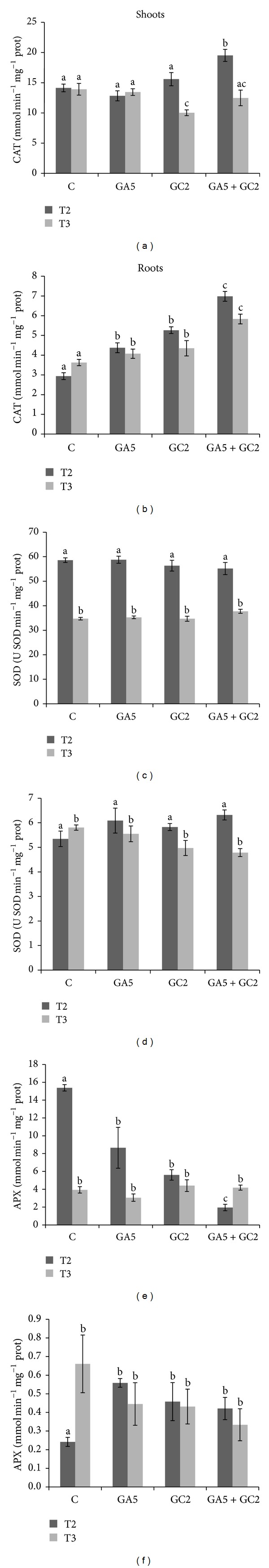
Enzyme activities in olive shoots (a, c, and e) and roots (b, d, and f). Catalase (CAT), superoxide dismutase (SOD), and ascorbate peroxidase (APX). Treatments: C (control without inoculation); GA5 (*Rhizophagus irregularis *GA5 strain inoculation); GC2 (*Rhizophagus irregularis *GC2 strain inoculation); GA5 + GC2 (mixture 1 : 1 of GA5 and GC2 strains). T2 (1-year plants on sterile soil); T3 (7 d after transplant to nonsterile soil). Different letters indicate significant differences at *P* < 0.05. Data were analyzed with factorial ANOVA. Data represent mean of 6 replicates ± standard error.

**Figure 4:**
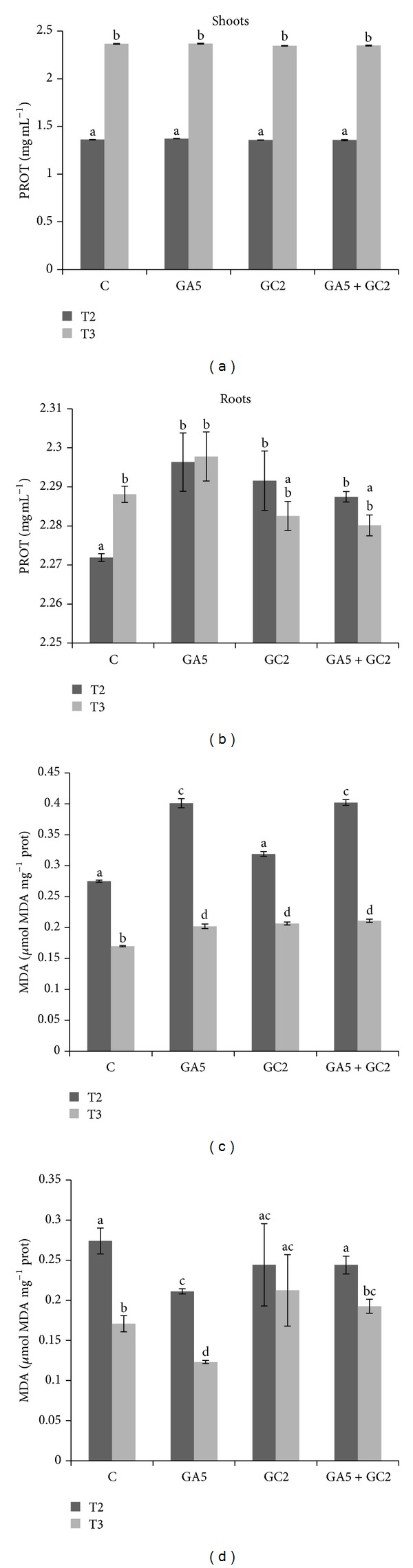
Total protein (PROT) and malondialdehyde (MDA) contents in olive shoots (a, c) and roots (b, d). Treatments: C (control without inoculation); GA5 (*Rhizophagus irregularis *GA5 strain inoculation); GC2 (*Rhizophagus irregularis *GC2 strain inoculation); GA5 + GC2 (mixture 1 : 1 of GA5 and GC2 strains). T2 (1-year plants on sterile soil); T3 (7 d after transplant to nonsterile soil). Different letters indicate significant differences at *P* < 0.05. Data were analyzed with factorial ANOVA. Data represent mean of 6 replicates ± standard error.

In olive shoot, the GC2-inoculated plants were found to have a decrease in CAT activity at T3 condition; coinoculated plants had higher CAT enzyme activity than control plants at T2; nevertheless, at T3 condition these values decreased at similar values compared to control plants at the same condition (Figure 3(a)). A decrease in SOD activity for all treatments was observed (Figure 3(c)). Inoculated plants had less APX enzyme activity at T2 condition than control plants. This difference was more markedly in coinoculated plants at T3 condition where they decreased at similar values compared to control plants (Figure 3(e)). PROT increased at T3 for all treatments (Figure 4(a)). The MDA content was higher in GA5 and coinoculated plants at T2 in comparison to control plants and GC2-inoculated ones but at T3 they decreased. However, these values were significantly higher than control plants (Figure 4(c)).

In roots coinoculated plants increased CAT enzyme activity at both conditions followed by single inoculation (Figure 3(b)). Inoculated plants decreased SOD enzyme activity at T3 in contrast to control plants (Figure 3(d)). No differences were observed in APX activity in inoculated plants at both conditions. Nevertheless these values were similar to control plants at T3 condition (Figure 3(f)). The same results were observed in PROT content (Figure 4(b)). The GA5-inoculated plants decreased MDA content at both conditions in comparison to control plants. There were no differences observed in MDA of GC2-inoculated plants at both conditions. However, these values were significantly higher than control plants at T3. Moreover, coinoculated plants had similar MDA content to control plants at both conditions assayed (Figure 4(d)).

## 4. Discussion

Olive plants inoculated with the GA5 strain and coinoculated improved survival at the first transplant condition. At the second transplant condition the improvement was due to coinoculation. Carpio et al. [31] in a study about commercial mycorrhiza under nursery and landscape conditions observed high survival of AMF plants in landscape condition. Thus the initial benefit of AMF inoculation should come in transplant establishment and growth. Mycorrhizal colonization was similar along the experiment; the proportion of arbuscules increased at the second transplant in GC2-inoculated plants whose slow growing behavior was observed *in vitro *and in soil in previous experiments [17]. At the first transplant olive plant growth was affected by transplant for all treatments; one year after, the GA5-inoculation improved growing before transplant but transplant response improvement was evidenced mainly by coinoculation followed by GA5-inoculation. Calvente et al. [32] found a growing improvement of Arbequina and Leccino olive cultivar cuttings with selected AMF species *R. irregularis *and *Glomus viscosum.* Also Porras-Soriano et al. [1] observed higher effectiveness of olive plants inoculated with *Funneliformis mosseae *compared to *R. irregularis *and *Claroideoglomus claroideum* against saline stress. The capability of AMF in protecting plants from the detrimental effects of salt stress may depend on the behaviour of each species.

At first transplant condition shoot and root water content decreased in all treatments. However, GA5-inoculation kept water in plants more efficiently than the GC2 and coinoculated plants at the second transplant condition. Alguacil et al. [33] found different levels of effectiveness in improving the growth of three shrub species with a mixture of native AMF being equal to or more effective than an allochthonous AMF. Mycorrhizal dependency was evidenced only at the second transplant condition for GC2 and coinoculated plants. Calvente et al. [32] observed an effectiveness of *R. irregularis *and *G. viscosum* in Arbequina olive cultivar followed by *G. mosseae *and *G. clarum*. On the other hand, Carpio et al. [34] found an increase of mycorrhizal dependency in a mixture of commercialized mycorrhizal inoculum compared to single inoculation. Coinoculation caused an additional growth cost but increased the survival of plants. At the second transplant olive growth was enhanced by coinoculation followed by GA5 strain inoculation.

In shoots SOD and APX enzyme activities were low under all treatments and transplant conditions applied; only CAT increased in GC2-inoculated plants at the first transplant. Alguacil et al. [33] found very low activity in all enzymes tested in nonmycorrhizal olive shoots. Moreover, they did not find differences in SOD activity but an increase in CAT enzyme activity in olive plants inoculated with *C. claroideum*. Therefore other mechanisms against adverse environmental conditions could also be involved such as nutrient uptake. Sofo et al. [35] observed an increase in SOD, APX, and CAT enzyme activities in Coratina olive cultivar without AMF inoculation. They also found an increase in MDA content during a progressive increment of drought stress. These results suggest that olive trees are able to upregulate the enzymatic antioxidant system. Our results show an increase in PROT content for all treatments and transplant tested. Moreover, a reduction in MDA content associated with a reduction in damage to lipids was observed at the first transplant condition. However at the second transplant condition they increased. Bacelar et al. [5] found an increase in lipid peroxidation in three olive cultivars without AMF inoculation in response to drought stress. They also found different levels of susceptibility to lipid peroxidation among the olive tree cultivars. In our experiments the activation of defense mechanisms to overcome transplant stress was observed at both transplant conditions with variations upon treatments.

In roots only SOD decreases in all treatments at the first transplant. Moreover, at the second transplant condition a decrease in SOD enzyme activity was observed in inoculated plants. These results are in concordance with Sofo et al. [35] who found a decrease in SOD enzyme activity in inoculated olive plants under abiotic stress. Ruíz-Lozano et al. [36] observed that mycorrhizal plants possess enhanced activity of several antioxidant enzymes but the response of the individual enzyme has been shown to vary with the fungal species and the host plant. On the other hand, our results show an increase in CAT enzyme activity in inoculated plants at the second transplant condition. This is in concordance with the observations of Wu et al. [37] in citrus roots inoculated with *Glomus versiforme *under water stress conditions who found that AMF plants show higher antioxidant enzymes during adverse conditions and the breakage of ROS is alleviated by AMF colonization. A reduction of lipid damage was observed at the first transplant condition for all treatments tested. However, at the second transplant condition this reduction was observed mainly in GA5-inoculated plants followed by coinoculated ones. Wu et al. [37] found high levels of MDA content in nonmycorrhizal citrus roots at water stress condition; thus mycorrhizal plants were more tolerant against drought stress than nonmycorrhizal ones.

Our results show a differential tolerance between the strains tested and transplant conditions. Marin [7] found that under nursery conditions the beneficial effects of AMF are less evident due to the controlled conditions; an early mycorrhizal inoculation may help to improve the transplant outside. In a previous experiment we observed a beneficial effect of AMF due to coinoculation under nursery conditions [38].

## 5. Conclusions

In conclusion, the first transplant resulted in an adaptative condition to the growing stage and to the mycorrhizal colonization causing an additional energetic expense in plants. However, in the second transplant the coinoculation improved olive plant growth and protected against oxidative stress followed by the GA5-inoculation. A combination of two AMF strains at the beginning of olive propagation produced vigorous plants successfully protected in field cultivation even with an additional cost at the beginning of plant growth.

## Figures and Tables

**Table 1:** Mycorrhizal colonization and survival percentages in olive plants at the first transplant condition.

Treatment	MI%	A%	V%	Survival%
C	n.d.	n.d.	n.d.	100.0 ± 0.001^a^
GA5	63.4 ± 7.54^a^	17.9 ± 2.89^b^	42.4 ± 7.74^c^	92.5 ± 1.59^ab^
GC2	61.0 ± 5.38^a^	28.9 ± 6.64^b^	43.3 ± 5.25^c^	87.5 ± 3.22^b^
GA5 + GC2	56.6 ± 5.05^a^	15.5 ± 4.89^b^	45.5 ± 6.94^c^	91.7 ± 2.15^ab^
ANOVA	n.s.	n.s.	n.s.	∗∗∗

Treatments: C (control without inoculation); GA5 (*Rhizophagus irregularis* GA5 strain inoculation); GC2 (*Rhizophagus irregularis* GC2 strain inoculation); GA5 + GC2 (mixture 1 : 1 of GA5 and GC2 strains). Mycorrhizal (MI%), arbuscules (A%), and vesicles (V%) percentages. Not detectable (n.d.). Different letters in the same column indicate significant differences at ***P < 0.001, not significant (n.s.). Data represent mean values of 12 replicates ± standard error.

**Table 2:** Growth parameters in olive plants on the first transplant condition.

Treatment	Fresh weight (g)		Biomass (g)		Water content (g H_2_O)		Shoot-to-root ratio	MD (%)	
	Shoots	Roots	Shoots	Roots	Shoots	Roots		Shoots	Roots
T0									
C	1.2 ± 0.14^a^	0.3 ± 0.06^a^	0.5 ± 0.06^a^	0.06 ± 0.001^a^	0.7 ± 0.13^a^	0.3 ± 0.04^a^	9.6 ± 1.53^a^		
GA5	1.6 ± 0.40^a^	0.5 ± 0.18^a^	0.7 ± 0.14^a^	0.09 ± 0.030^a^	1.0 ± 0.44^a^	0.4 ± 0.26^a^	13.7 ± 9.18^a^	125.9 ± 26.66^a^	158.5 ± 55.65^a^
GC2	1.7 ± 0.06^a^	0.6 ± 0.23^a^	0.7 ± 0.01^a^	0.1 ± 0.05^a^	1.0 ± 0.08^a^	0.4 ± 0.31^a^	15.7 ± 10.34^a^	135.0 ± 2.61^a^	185.1 ± 90.33^a^
GA5 + GC2	1.6 ± 0.24^a^	0.3 ± 0.18^a^	0.7 ± 0.10^a^	0.05 ± 0.030^a^	0.9 ± 0.23^a^	0.3 ± 0.26^a^	31.5 ± 14.19^a^	152.1 ± 2.58^a^	91.7 ± 59.05^a^
T1									
C	0.8 ± 0.06^b^	0.4 ± 0.04^a^	0.4 ± 0.01^b^	0.07 ± 0.001^a^	0.4 ± 0.10^b^	0.3 ± 0.07^a^	5.0 ± 0.15^a^		
GA5	1.2 ± 0.31^b^	0.4 ± 0.23^a^	0.5 ± 0.13^b^	0.07 ± 0.040^a^	0.7 ± 0.31^b^	0.3 ± 0.32^a^	11.2 ± 3.60^a^	132.2 ± 36.52^a^	98.5 ± 62.09^a^
GC2	0.7 ± 0.03^b^	0.4 ± 0.15^a^	0.3 ± 0.02^b^	0.07 ± 0.001^a^	0.4 ± 0.02^b^	0.3 ± 0.25^a^	4.4 ± 0.70^a^	82.1 ± 5.74^a^	95.7 ± 9.92^a^
GA5 + GC2	1.2 ± 0.21^b^	0.2 ± 0.07^a^	0.5 ± 0.09^b^	0.04 ± 0.010^a^	0.7 ± 0.20^b^	0.2 ± 0.09^a^	13.6 ± 3.67^a^	134.2 ± 24.14^a^	60.2 ± 19.81^a^

ANOVA									
T	∗	n.s.	∗	n.s.	∗	n.s.	n.s.	n.s.	n.s.
AMF	n.s.	n.s.	n.s.	n.s.	n.s.	n.s.	n.s.	n.s.	n.s.
T × AMF	n.s.	n.s.	n.s.	n.s.	n.s.	n.s.	n.s.	n.s.	n.s.

Treatments: C (control without inoculation); GA5 (*Rhizophagus irregularis* GA5 strain inoculation); GC2 (*Rhizophagus irregularis* GC2 strain inoculation); GA5 + GC2 (mixture 1 : 1 of GA5 and GC2 strains); MD (mycorrhizal dependency); T0 (on raised table); T1 (3 d transplanted on sterile soil). Different letters in the same column indicate significant differences at *P < 0.01, not significant (n.s.). Data represent mean values of 6 replicates ± standard error.

**Table 3:** Mycorrhizal colonization and survival percentages in olive plants at the second transplant condition.

Treatment	MI%	A%	V%	Survival%
C	n.d.	n.d.	n.d.	92.8 ± 0.05^a^
GA5	76.7 ± 5.23^a^	40.7 ± 7.60^a^	56.3 ± 6.78^a^	91.7 ± 0.03^a^
GC2	70.0 ± 5.85^a^	66.5 ± 5.48^b^	39.3 ± 6.48^a^	90.8 ± 2.63^a^
GA5 + GC2	67.2 ± 7.31^a^	45.9 ± 6.02^ab^	46.8 ± 8.62^a^	100.0 ± 0.001^b^
ANOVA	n.s.	∗∗∗	n.s.	∗∗∗

Treatments: C (control without inoculation); GA5 (*Rhizophagus irregularis* GA5 strain inoculation); GC2 (*Rhizophagus irregularis* GC2 strain inoculation); GA5 + GC2 (mixture 1 : 1 of GA5 and GC2 strains). Mycorrhizal (MI%), arbuscules (A%), and vesicles (V%) percentages. Not detectable (n.d.). Different letters in the same column indicate significant differences at ***P < 0.001, not significant (n.s.). Data represent mean values of 12 replicates ± standard error.

**Table 4:** Growth parameters in olive plants at the second transplant condition.

Treatment	Fresh weight (g)		Biomass (g)		Water content (g H_2_O)		Shoot-to-root ratio	MD (%)	
	Shoots	Roots	Shoots	Roots	Shoots	Roots		Shoots	Roots
T2									
C	1.4 ± 0.49^a^	0.6 ± 0.19^a^	0.6 ± 0.22^a^	0.2 ± 0.05^a^	0.8 ± 0.27^a^	0.4 ± 0.14^a^	3.2 ± 0.81^a^		
GA5	3.1 ± 0.63^b^	3.9 ± 1.37^b^	1.2 ± 0.27^b^	1.1 ± 0.45^b^	1.9 ± 0.36^b^	2.8 ± 0.92^b^	1.3 ± 0.31^a^	204 ± 46.3^a^	573 ± 238.5^a^
GC2	1.0 ± 0.23^a^	0.5 ± 0.13^a^	0.4 ± 0.06^a^	0.2 ± 0.04^a^	0.6 ± 0.18^c^	0.3 ± 0.15^a^	2.4 ± 0.70^a^	63 ± 11.3^b^	94 ± 23.9^b^
GA5 + GC2	0.9 ± 0.15^a^	0.5 ± 0.10^a^	0.3 ± 0.06^a^	0.1 ± 0.03^a^	0.5 ± 0.09^c^	0.4 ± 0.06^a^	2.7 ± 0.18^a^	55 ± 10.4^b^	66 ± 16.2^b^
T3									
C	0.7 ± 0.13^a^	0.3 ± 0.11^a^	0.5 ± 0.05^a^	0.2 ± 0.01^a^	0.3 ± 0.09^c^	0.2 ± 0.02^a^	2.3 ± 0.40^a^		
GA5	1.3 ± 0.53^a^	1.5 ± 1.01^a^	0.7 ± 0.25^a^	0.7 ± 0.45^b^	0.7 ± 0.27^ac^	0.8 ± 0.55^c^	1.7 ± 0.54^a^	139 ± 54.4^c^	322 ± 216.1^c^
GC2	1.8 ± 0.17^a^	1.2 ± 0.12^a^	0.8 ± 0.08^a^	0.5 ± 0.05^b^	0.9 ± 0.09^a^	0.7 ± 0.07^c^	1.5 ± 0.03^a^	174 ± 17.6^c^	254 ± 26.1^c^
GA5 + GC2	2.7 ± 0.51^b^	2.2 ± 0.91^b^	1.3 ± 0.27^b^	0.9 ± 0.37^b^	1.4 ± 0.24^ab^	1.3 ± 0.54^c^	1.8 ± 0.44^a^	237 ± 68.7^a^	435 ± 175.4^a^

ANOVA									
T	n.s.	n.s.	n.s.	n.s.	n.s.	n.s.	n.s.	∗	∗
AMF	∗	∗	n.s.	n.s.	∗	∗∗	n.s.	n.s.	n.s.
T × AMF	∗∗∗	∗	∗	n.s.	∗∗∗	∗	n.s.	∗	∗

Treatments: C (control without inoculation); GA5 (*Rhizophagus irregularis* GA5 strain inoculation); GC2 (*Rhizophagus irregularis* GC2 strain inoculation); GA5 + GC2 (mixture 1 : 1 of GA5 and GC2 strains); MD (mycorrhizal dependency); T2 (1-year plants on sterile soil); T3 (7 d after transplant to nonsterile soil). Different letters in the same column indicate significant differences at *P < 0.05, **P < 0.01, ***P < 0.001, not significant (n.s.). Data represent mean of 6 replicates ± standard error.
